# The impact of magnesium sulfate administration on outcomes in ICU patients with acute myocardial infarction: a retrospective cohort study

**DOI:** 10.3389/fphar.2026.1776926

**Published:** 2026-05-25

**Authors:** Guibao Jiang, Gangzhi Zhuang, Hui Zhao, Rui Su, Xin Niu, Liqiong Wang, Jiayu Wu, Jia Liu, Jianchuan Zhang, Wengui Li, Qilin Yang, Rong Ding

**Affiliations:** 1 Department of Critical Care Medicine, Huian County Hospital, Quanzhou, Fujian, China; 2 Department of Pulmonary and Critical Care Medicine, Second Hospital of Shanxi Medical University, Taiyuan, Shanxi, China; 3 Department of Respiratory Intensive Care Unit, Second Hospital of Shanxi Medical University, Taiyuan, Shanxi, China; 4 Department of Critical Care, The Second Affiliated Hospital of Guangzhou Medical University, Guangzhou, Guangdong, China

**Keywords:** acute myocardial infarction, intensive care unit, magnesium sulfate, mortality, propensity score matching

## Abstract

**Background:**

Although magnesium acts as a natural calcium antagonist with theoretical cardioprotective effects, its clinical value in critically ill patients with acute myocardial infarction (AMI) remains controversial. This study aimed to evaluate the impact of intravenous magnesium sulfate on ICU mortality in this population, and to explore potential dose-response relationships and effect modifiers.

**Methods:**

This retrospective cohort study utilized the single-center Medical Information Mart for Intensive Care-IV version 3.1 (MIMIC-IV v3.1) database, which comprises patients admitted to Beth Israel Deaconess Medical Center, to examine the association between intravenous magnesium sulfate administration and mortality in critically ill patients with AMI. The primary endpoint was ICU mortality. Multivariable Cox regression, propensity score matching, and propensity score weighting were employed to control for confounders and estimate the independent effect of magnesium sulfate administration. Additional analyses included assessment of the restricted mean survival time (RMST) and quantitative evaluation of unmeasured confounding using the E-value.

**Results:**

This study included 5,650 critically ill patients with AMI. In the propensity score-matched analysis, intravenous magnesium sulfate administration was associated with a 45% reduction in ICU mortality risk (HR 0.55, 95% CI 0.42–0.71). RMST analysis confirmed an absolute survival benefit of 1.64 days at 14 days. A consistent reduction in mortality risk was observed across all secondary endpoints. A clear dose-response relationship was identified, with the greatest survival benefit at moderate cumulative doses (2.0–8.0 g) and treatment durations (1.08–3.21 days). The association remained robust to potential unmeasured confounding (E-value = 2.66). Subgroup analyses demonstrated consistent protective effects across all patient strata.

**Conclusion:**

Intravenous magnesium sulfate administration is associated with lower ICU mortality in critically ill patients with AMI.

## Graphical Abstract

**Figure:**
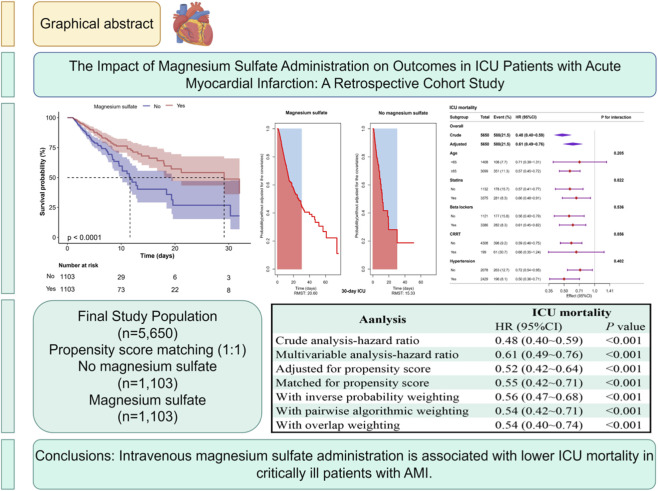

## Introduction

Acute Myocardial Infarction (AMI) remains a leading cause of global mortality, with ischemic heart disease accounting for over 9 million deaths annually according to the 2019 Global Burden of Disease Study (Roth et al., 2020; Vaduganathan et al., 2022). Despite significant advances in reperfusion therapy and percutaneous coronary intervention, critically ill AMI patients admitted to the intensive care unit remain at high risk of adverse outcomes. Those presenting with cardiogenic shock experience particularly high mortality, with reported rates of 40% at 30 days and nearly 50% at 1 year (Samsky et al., 2021; Jortveit et al., 2025). These individuals frequently exhibit hemodynamic instability, severe arrhythmias, acute heart failure, or multi-organ dysfunction, creating an urgent need for novel therapeutic strategies to mitigate mortality risk and enhance long-term survival (Chioncel et al., 2020; Thiele et al., 2023).

Magnesium, the fourth most abundant cation and the second most prevalent intracellular cation in the human body, participates in over 600 enzymatic reactions and plays critical roles in maintaining myocardial electrophysiological stability, vascular tone, and energy metabolism (De Baaij et al., 2015; Kröse and De Baaij, 2024). Magnesium deficiency is frequently observed in AMI patients, with reported prevalence rates ranging from 30% to 60%, and has been closely associated with increased risk of ventricular arrhythmias (Kafka et al., 1987; Hasan et al., 2023). Pathophysiological studies demonstrate that magnesium deficiency can lead to intracellular calcium overload, activate oxidative stress responses, promote platelet aggregation, and exacerbate myocardial ischemia-reperfusion injury (Negru et al., 2022). Magnesium sulfate supplementation may confer cardioprotective effects through multiple mechanisms: functioning as a natural calcium channel antagonist to inhibit calcium influx and mitigate calcium-mediated cardiomyocyte injury (Garcia et al., 1998; Matsusaka et al., 2002); suppressing catecholamine release, improving endothelial function, and reducing free radical generation to alleviate reperfusion injury (Herzog et al., 1995).

Early clinical interest in intravenous magnesium sulfate for AMI was bolstered by the Leicester Intravenous Magnesium Intervention Trial (LIMIT-2). Published in *The Lancet* in 1992, this randomized trial enrolled 2,316 patients and reported a significant reduction in 28-day mortality with early administration of a total magnesium sulfate dose of 73 mmol (approximately 17.7 g), given as an initial 8 mmol bolus followed by 65 mmol over 24 h (Woods et al., 1992; Woods and Fletcher, 1994). However, this finding was not corroborated by the subsequent large-scale Fourth International Study of Infarct Survival (ISIS-4), which included 58,050 patients administered a total dose of 80 mmol (approximately 20 g) over 24 h, finding no mortality benefit and suggesting a potential trend toward increased early mortality (Clinical Trial, 1995). More recent evidence, including a 2021 systematic review, indicates that administration timing—particularly around reperfusion—may influence outcomes, such as reducing reperfusion arrhythmias (Baker, 2016; Szapary et al., 2021). Despite this, evidence regarding the effect of magnesium sulfate specifically in critically ill AMI patients admitted to the intensive care unit remains notably limited.

This study aimed to assess the association between intravenous magnesium sulfate therapy and mortality in critically ill AMI patients admitted to the ICU. Data were derived from the Medical Information Mart for Intensive Care-IV version 3.1 (MIMIC-IV v3.1) database, a single-center critical care database from Beth Israel Deaconess Medical Center. We also examined dose-response relationships for cumulative dosage and treatment duration with key clinical outcomes to generate quantitative evidence for therapeutic guidance.

## Materials and methods

### Data source

This retrospective cohort study utilized data from the Medical Information Mart for Intensive Care IV (MIMIC-IV, v3.1), which contains de-identified clinical records of ICU patients at Beth Israel Deaconess Medical Center (Boston, MA, United States) from 2008 to 2022 (Johnson et al., 2023). Ethical approval with a waiver of informed consent was granted by the Institutional Review Boards of both MIT and Beth Israel Deaconess Medical Center. Author Rong Ding completed the required NIH human research protection course (Certification No. 64760223) for database access. This study adhered to the STROBE guidelines and the Declaration of Helsinki (Cuschieri, 2019).

### Study population

**FIGURE 1:**
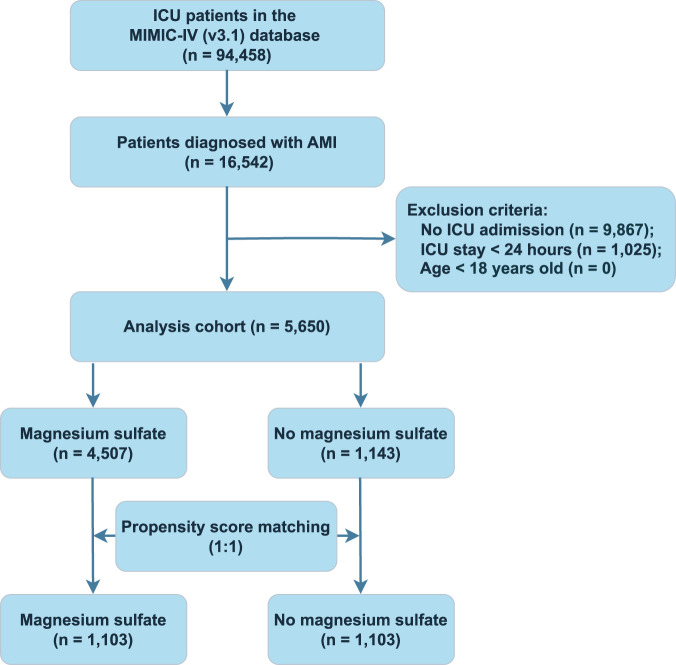
Flowchart of the study cohort. ICU, intensive care unit; MIMIC-IV, medical information Mart for intensive care IV.

A total of 16,542 adult patients with AMI were initially identified from the database using relevant International Classification of Diseases (ICD)-9 and ICD-10 codes. The exclusion criteria were: (1) no ICU admission (n = 9,867); and (2) ICU length of stay less than 24 h (n = 1,025). After sequential screening, 5,650 eligible patients were included in the final cohort. Based on intravenous magnesium sulfate administration during the ICU stay, patients were categorized into the magnesium sulfate group (n = 4,507; 79.8%) and the no magnesium sulfate group (n = 1,143; 20.2%), as shown in Figure 1.

### Demographic and laboratory variables

Data were extracted from the MIMIC-IV database using Structured Query Language (SQL). The collected parameters included: (1) demographic characteristics: age, sex, and hypertension history; (2) vital signs: mean arterial pressure, temperature, and oxygen saturation (SpO_2_); (3) laboratory measurements: electrolytes (magnesium, potassium, calcium, sodium), hemoglobin, platelet count, creatinine, prothrombin time, and lactate; (4) illness severity score: the Sequential Organ Failure Assessment (SOFA); and (5) treatments and procedures: anticoagulants, statins, beta-blockers, vasoactive agents, mechanical ventilation, percutaneous coronary intervention (PCI), continuous renal replacement therapy (CRRT), and extracorporeal membrane oxygenation (ECMO). All laboratory parameters were defined as the first measured values within 24 h of ICU admission. Missing data details for each variable are presented in Supplementary Table S2. To address missingness, we employed multiple imputation using chained equations with the R mice package, with the imputation model including all variables listed above. Five complete datasets were generated, and analyses were performed separately on each dataset, with results combined using Rubin’s rules to obtain pooled estimates.

### Exposure and outcomes

The primary exposure was the administration of intravenous magnesium sulfate during the ICU stay. Medication records were reviewed to extract information on magnesium sulfate use, including cumulative dosage and duration of treatment. In this cohort, magnesium sulfate was administered as part of a cardiac protocol for arrhythmia prophylaxis and myocardial protection. These data were subsequently used for stratified analyses. For dose-response analysis, patients were stratified into three subgroups by cumulative dose: < 2.0 g, 2.0–8.0 g, and ≥ 8.0 g; as well as by treatment duration: < 1.08 days, 1.08–3.21 days, and ≥ 3.21 days.

The primary outcome was ICU mortality. Secondary outcomes comprised in-hospital mortality, all-cause mortality at 30, 60, 90, and 365 days, and the counts of vasopressor-free, ICU-free, and ventilator-free days within 28 days following ICU admission. Mortality data were obtained from medical records and the U.S. Social Security Administration Death Master File, with follow-up censored at the date of death or 31 December 2019, whichever occurred first.

### Statistical analysis

Continuous variables were expressed as mean ± standard deviation (SD) or median and interquartile range (IQR), as appropriate, based on their distribution. Categorical variables were reported as numbers (percentages). The balance of baseline characteristics between groups was evaluated using standardized mean differences (SMD), with an SMD of less than 0.1 indicating a negligible imbalance.

**FIGURE 2:**
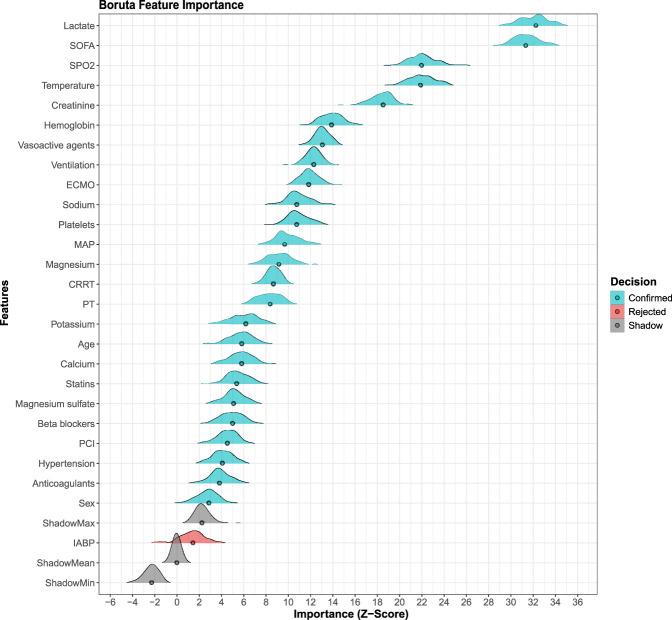
Feature selection using the Boruta algorithm. Variable names are listed on the vertical axis, and their importance (Z-score) is shown on the horizontal axis. Each boxplot depicts the distribution of Z-scores for a variable. Green boxes indicate important variables selected for the propensity score model; red boxes indicate unimportant variables that were rejected. SOFA, Sequential Organ Failure Assessment; ECMO, Extracorporeal Membrane Oxygenation; MAP, Mean Arterial Pressure; CRRT, Continuous Renal Replacement Therapy; PT, Prothrombin Time; PCI, Percutaneous Coronary Intervention; IABP, Intra-Aortic Balloon Pump.

To address potential confounding, we performed propensity score matching (PSM) to balance baseline characteristics between the groups, based on covariates that were selected through a data-driven process using the Boruta algorithm for all-relevant feature selection. All available baseline variables were run through the Boruta algorithm, and those confirmed as important were included in the propensity score model. The selected variables were: age, hypertension, mean arterial pressure, temperature, SpO_2_, magnesium, potassium, calcium, sodium, hemoglobin, creatinine, platelet count, prothrombin time, lactate, and the use of anticoagulants, statins, beta-blockers, vasoactive agents, mechanical ventilation, PCI, CRRT, ECMO, as well as the SOFA score. The results of this feature selection process are presented in Figure 2. Subsequently, a 1:1 nearest-neighbor matching algorithm was applied with a caliper width of 0.2, resulting in 1,103 well-matched pairs for subsequent analysis.

Survival analyses were conducted using the Kaplan-Meier method, and between-group differences across all predefined time-to-event mortality endpoints were compared with the log-rank test. To complement hazard ratio estimates and quantify the absolute survival difference, the restricted mean survival time (RMST) was also calculated and compared between groups over 14-day and 30-day intervals.

The association between intravenous magnesium sulfate treatment and mortality risk was evaluated using Cox proportional hazards models. To assess the robustness of the findings, multiple analytical approaches were applied, including: (1) an unmatched crude model; (2) a multivariable-adjusted model; (3) a propensity score-adjusted model; (4) a propensity score-matched model; (5) inverse probability of treatment weighting (IPTW) (Bettega et al., 2024); (6) pairwise algorithmic (PA), and (7) overlap weighting (OW). Results are reported as hazard ratios (HR) with 95% confidence intervals (CIs). Additionally, multivariable linear regression was employed to evaluate the treatment effects on 28-day organ support-free days, with results presented as beta coefficients (β) and 95% CIs.

Prespecified subgroup analyses were performed to explore potential effect heterogeneity. Subgroups were defined by age (< 65 vs. ≥ 65 years), history of hypertension, and use of CRRT, beta-blockers, or statins. Interaction effects were evaluated using the likelihood ratio test, with a p-value for interaction of less than 0.05 considered statistically significant.

For the dose-response analysis, the lowest dose group (< 2.0 g) and the shortest duration group (< 1.08 days) were set as reference categories. Treatment effects across different dosage and duration strata were evaluated using multivariable-adjusted Cox proportional hazards models. To further validate the stability of the dose-response findings, we additionally performed propensity score matching across the three dosage groups. As a sensitivity analysis, we calculated the E-value for both the point estimate and the upper confidence limit of the hazard ratio for the primary outcome to evaluate the potential impact of unmeasured confounding on the observed associations.

All analysis was performed using R 4.2.2 (http://www.Rproject.org; The R Foundation, Vienna, Austria) and the Free Statistics software (version 2.2; Beijing FreeClinical Medical Technology Co., Ltd, Beijing, China). Statistical significance was indicated by *P* < 0.05.

## Results

### Baseline characteristics of study subjects

The baseline characteristics of the total cohort are presented in Supplementary Table S1. Among the 5,650 eligible patients, the mean age was 71.1 years, 64.7% were male, and the overall ICU mortality rate was 10.4%. The baseline characteristics of patients before and after propensity score matching are presented in Table 1. Prior to matching, patients in the magnesium sulfate group were generally younger, had a higher prevalence of hypertension, and demonstrated lower serum calcium and creatinine levels along with reduced platelet counts. Additionally, they more frequently received statins (Table 1), beta-blockers, vasoactive agents, and mechanical ventilation, and presented with higher SOFA scores, collectively suggestive of a greater critical illness burden at baseline.

**TABLE 1:** Imbalance of critically ill patients with AMI characteristics before and after propensity score matching in the assessment of ICU mortality.

Variables	Before propensity score matching			After propensity score matching		
	No magnesium sulfate (n = 1143)	Magnesium sulfate (n = 4507)	SMD	No magnesium sulfate (n = 1103)	Magnesium sulfate (n = 1103)	SMD
Age (years)	73.38 (13.55)	70.56 (12.72)	0.215	73.26 (13.54)	73.63 (12.92)	0.028
Male (n, %)	726 (63.50)	2928 (65.00)	0.030	705 (63.90)	702 (63.60)	0.006
Hypertension (n, %)	503 (44.00)	2429 (53.90)	0.199	490 (44.40)	495 (44.90)	0.009
MAP (mmHg)	77.53 (11.11)	77.44 (9.50)	0.009	77.54 (11.00)	77.91 (10.01)	0.035
Temperature (°C)	36.74 (0.49)	36.80 (0.54)	0.122	36.74 (0.49)	36.74 (0.51)	0.013
SpO_2_ (%)	96.63 (1.81)	96.97 (2.07)	0.175	96.64 (1.81)	96.59 (2.07)	0.024
Magnesium (mmol/L)	2.18 (0.36)	2.11 (0.52)	0.153	2.17 (0.35)	2.14 (0.55)	0.064
Potassium (mmol/L)	4.84 (0.93)	4.73 (0.81)	0.129	4.81 (0.90)	4.83 (0.95)	0.013
Calcium (mmol/L)	8.36 (0.77)	8.15 (0.77)	0.283	8.34 (0.77)	8.35 (0.75)	0.005
Hemoglobin (g/dL)	10.33 (2.44)	10.02 (2.26)	0.133	10.36 (2.44)	10.42 (2.37)	0.025
Sodium (mmol/L)	136.52 (5.11)	136.27 (4.63)	0.052	136.53 (5.12)	136.50 (5.07)	0.005
Creatinine (mg/dL)	2.44 (2.61)	1.67 (1.60)	0.355	2.30 (2.18)	2.20 (2.40)	0.045
Platelets (10^9^/L)	199.39 (88.51)	180.30 (85.65)	0.219	199.07 (88.67)	199.00 (91.75)	0.001
PT (s)	17.03 (11.52)	16.90 (9.81)	0.012	16.91 (11.08)	16.69 (10.79)	0.020
Lactate (mmol/L)	2.65 (1.74)	2.96 (2.26)	0.153	2.66 (1.76)	2.72 (1.78)	0.037
Anticoagulants (n, %)	1023 (89.50)	3831 (85.00)	0.135	988 (89.60)	999 (90.60)	0.033
Statins (n, %)	616 (53.90)	3375 (74.90)	0.449	603 (54.70)	598 (54.20)	0.009
Beta blockers (n, %)	777 (68.00)	3386 (75.10)	0.159	753 (68.30)	760 (68.90)	0.014
Vasoactive agents (n, %)	251 (22.00)	2033 (45.10)	0.506	246 (22.30)	247 (22.40)	0.002
Ventilation (n, %)	228 (19.90)	2082 (46.20)	0.581	228 (20.70)	214 (19.40)	0.032
PCI (n, %)	282 (24.70)	790 (17.50)	0.176	278 (25.20)	284 (25.70)	0.012
CRRT (n, %)	99 (8.70)	199 (4.40)	0.172	88 (8.00)	89 (8.10)	0.003
ECMO (n, %)	3 (0.30)	19 (0.40)	0.027	3 (0.30)	3 (0.30)	<0.001
SOFA (score)	4.46 (3.36)	5.30 (3.31)	0.251	4.44 (3.39)	4.38 (3.20)	0.019

An absolute SMD < 10% was considered to support the assumption of a balance between the groups. Data are presented as medians [interquartile ranges], mean [SD] or as numbers (percentages).

Abbreviations: AMI, acute myocardial infarction; ICU, intensive care unit; SMD, standardized mean difference; MAP, mean arterial pressure; SpO_2_, pulse oxygen saturation; PT, prothrombin time; PCI, percutaneous coronary intervention; CRRT, continuous renal replacement therapy; ECMO, extracorporeal ane oxygenation; SOFA, sequential organ failure assessment.

**FIGURE 3:**
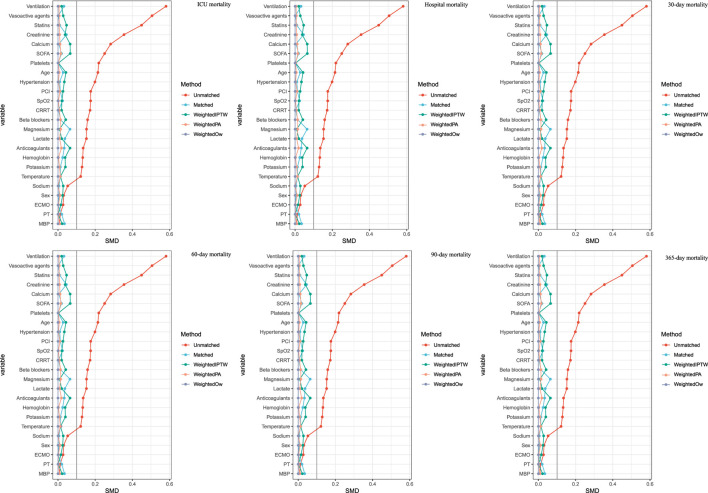
Standardized mean differences (SMD) of covariates before and after propensity score matching across six mortality endpoints.

The propensity score model exhibited good discriminative power, with an area under the receiver operating characteristic curve of 0.828 (95% CI: 0.810–0.846) (Supplementary Figure S1). After matching, all covariates were well balanced between the two groups, as visually confirmed by the Standardized Mean Difference plot (Figure 3), with all SMD values below 0.1, indicating successful balancing of baseline characteristics and supporting the validity of subsequent comparative analyses.

### Kaplan-Meier survival analysis

**FIGURE 4:**
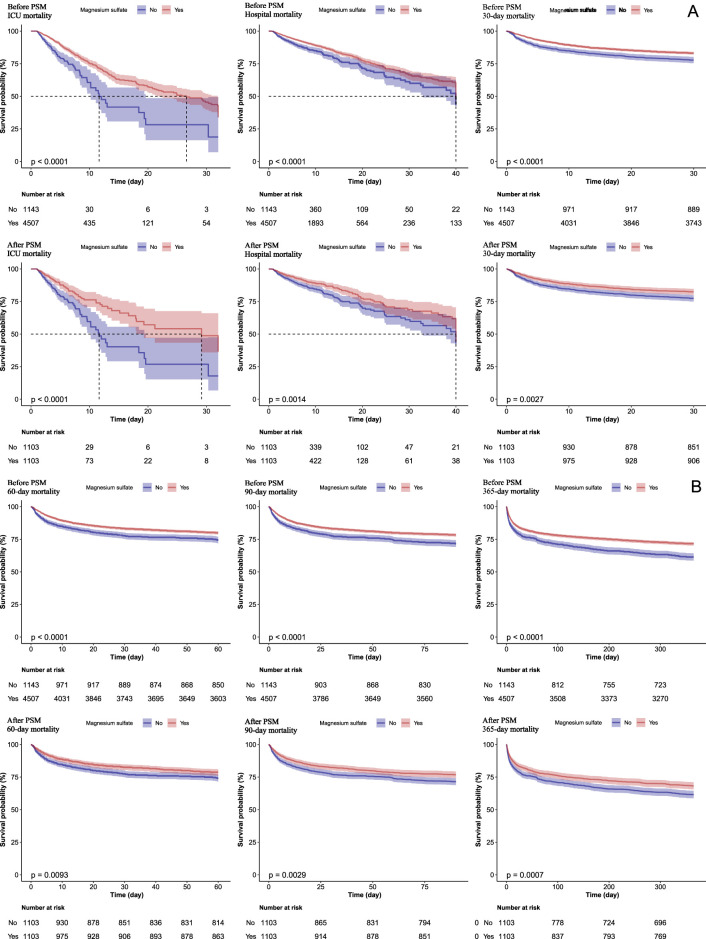
**(A)** Kaplan-Meier survival curves for ICU, in-hospital, and 30-day mortality before and after propensity score matching. **(B)** Kaplan-Meier survival curves for 60-day, 90-day, and 365-day mortality before and after propensity score matching.

Figure 4 show the Kaplan-Meier survival curves for the six mortality endpoints before and after propensity score matching. In the pre-matched cohorts, patients receiving magnesium sulfate demonstrated significantly superior survival across all endpoints (all log-rank *P* < 0.0001). After matching, the survival benefit remained statistically significant for the primary outcome of ICU mortality (log-rank *P* < 0.0001) and all secondary mortality endpoints (all log-rank *P* < 0.01).

### Restricted mean survival time analysis

**FIGURE 5:**
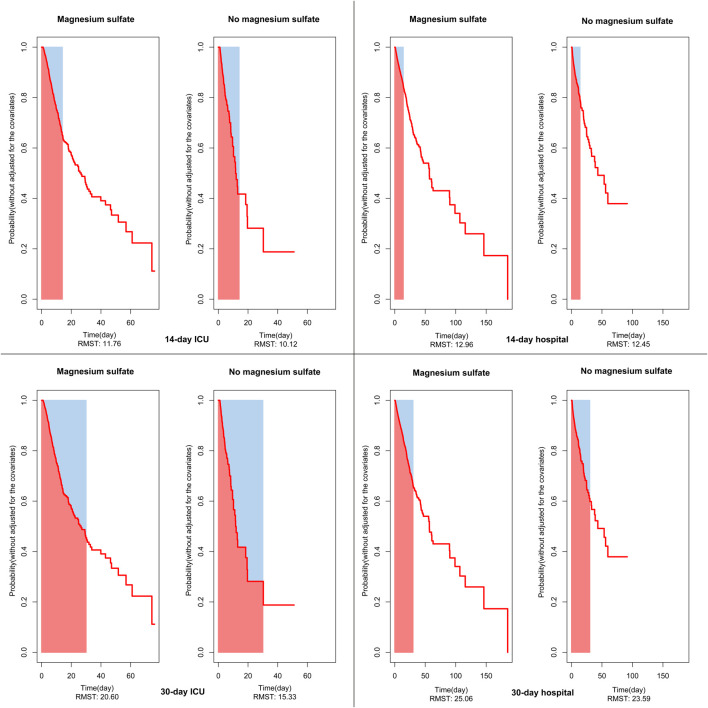
Restricted mean survival time (RMST) analysis for ICU and hospital mortality.

To complement hazard ratio analyses and quantify the absolute survival benefit, we evaluated the treatment effect using the difference in Restricted Mean Survival Time (RMSTd) over 14-day and 30-day intervals (Supplementary Table S3; Figure 5). For the primary outcome of ICU mortality, patients receiving magnesium sulfate had a significantly longer mean survival time within both time windows. At 14 days, the RMST was 11.76 days in the magnesium sulfate group compared to 10.12 days in the control group, corresponding to an absolute gain of 1.64 days (95% CI, 0.97–2.31; P < 0.001). The benefit was more pronounced over the 30-day window, with an RMST of 20.60 days versus 15.33 days in controls, resulting in a mean survival time difference of 5.27 days (95% CI, 2.63–7.91; P < 0.001). A similar trend of increased RMST was observed for hospital mortality.

### Association of magnesium sulfate with clinical outcomes

The associations between magnesium sulfate treatment and mortality across different time points under various statistical models are summarized in Table 2. In the unmatched crude analysis, magnesium sulfate administration was associated with a significant 52% reduction in ICU mortality risk (HR 0.48, 95% CI 0.40–0.59, *P* < 0.001). This association remained statistically significant after multivariable adjustment (HR 0.61, 95% CI 0.49–0.76, *P* < 0.001) and in propensity score-adjusted analyses (HR 0.52, 95% CI 0.42–0.64, *P* < 0.001). The propensity score-matched cohort demonstrated a 45% lower risk of ICU mortality in the magnesium sulfate group (HR 0.55, 95% CI 0.42–0.71, *P* < 0.001). Similar associations were observed with inverse probability of treatment weighting (HR 0.56, 95% CI 0.47–0.68, *P* < 0.001), pairwise algorithmic weighting (HR 0.54, 95% CI 0.42–0.71, *P* < 0.001), and overlap weighting approaches (HR 0.54, 95% CI 0.40–0.74, *P* < 0.001).

**TABLE 2:** Association between intravenous magnesium sulfate administration and mortality in critically ill patients with acute myocardial infarction using different analytical models.

Aanlysis	ICU mortality		Hospital mortality		30-day mortality		60-day mortality		90-day mortality		365-day mortality	
	HR (95% CI)	*P* value	HR (95% CI)	*P* value	HR (95% CI)	*P* value	HR (95% CI)	*P* value	HR (95% CI)	*P* value	HR (95% CI)	*P* value
Crude analysis-hazard ratio	0.48 (0.40–0.59)	<0.001	0.71 (0.60–0.83)	<0.001	0.73 (0.63–0.84)	<0.001	0.75 (0.65–0.85)	<0.001	0.73 (0.64–0.83)	<0.001	0.69 (0.62–0.77)	<0.001
Multivariable analysis-hazard ratio	0.61 (0.49–0.76)	<0.001	0.77 (0.64–0.92)	0.003	0.77 (0.66–0.90)	0.001	0.80 (0.69–0.92)	0.002	0.78 (0.68–0.90)	0.001	0.79 (0.70–0.89)	<0.001
Adjusted for propensity score	0.52 (0.42–0.64)	<0.001	0.72 (0.60–0.86)	<0.001	0.79 (0.68–0.93)	0.003	0.82 (0.71–0.95)	0.009	0.81 (0.71–0.94)	0.004	0.82 (0.73–0.92)	0.001
Matched for propensity score	0.55 (0.42–0.71)	<0.001	0.71 (0.58–0.89)	0.002	0.77 (0.64–0.93)	0.006	0.81 (0.68–0.96)	0.017	0.79 (0.67–0.94)	0.007	0.80 (0.70–0.92)	0.002
With inverse probability weighting	0.56 (0.47–0.68)	<0.001	0.75 (0.64–0.88)	0.017	0.71 (0.62–0.82)	<0.001	0.73 (0.64–0.83)	<0.001	0.73 (0.64–0.83)	<0.001	0.75 (0.67–0.83)	<0.001
With pairwise algorithmic weighting	0.54 (0.42–0.71)	<0.001	0.74 (0.60–0.92)	0.001	0.83 (0.69–1.00)	0.018	0.87 (0.73–1.03)	0.054	0.85 (0.72–1.00)	0.022	0.85 (0.73–0.98)	0.007
With overlap weighting	0.54 (0.40–0.74)	<0.001	0.73 (0.57–0.94)	0.001	0.80 (0.64–1.00)	0.005	0.83 (0.68–1.02)	0.012	0.82 (0.67–1.00)	0.005	0.82 (0.70–0.97)	0.001

Adjusted for age, sex, hypertension, MAP, temperature, SpO_2_, magnesium, potassium, calcium, hemoglobin, sodium, creatinine, platelets, PT, lactate, anticoagulants, statins, beta blockers, vasoactive agents, ventilation, PCI, CRRT, ECMO, and SOFA.

Abbreviations: AMI, acute myocardial infarction; ICU, intensive care unit; MAP, mean arterial pressure; SpO_2_, pulse oxygen saturation; PT, prothrombin time; PCI, percutaneous coronary intervention; CRRT, continuous renal replacement therapy; ECMO, extracorporeal membrane oxygenation; SOFA, sequential organ failure assessment.

For secondary outcomes, magnesium sulfate treatment was associated with reduced mortality risks across all time points in the propensity score-matched analysis: hospital mortality (29% reduction; HR 0.71, 95% CI 0.58–0.89, *P* = 0.002), 30-day mortality (23% reduction; HR 0.77, 95% CI 0.64–0.93, *P* = 0.006), 60-day mortality (19% reduction; HR 0.81, 95% CI 0.68–0.96, *P* = 0.017), 90-day mortality (21% reduction; HR 0.79, 95% CI 0.67–0.94, *P* = 0.007), and 365-day mortality (20% reduction; HR 0.80, 95% CI 0.70–0.92, *P* = 0.002). These associations were consistently observed across multiple analytical approaches.

Multivariable linear regression analyses further evaluated the association between magnesium sulfate treatment and organ support-free days within 28 days (Table 3). In the fully adjusted model, magnesium sulfate administration was significantly associated with an increase of 1.13 days in vasopressor-free days (β = 1.13, 95% CI 0.57–1.69, *P* < 0.001), an increase of 1.17 days in ICU-free days (β = 1.17, 95% CI 0.63–1.71, *P* < 0.001), and an increase of 1.26 days in ventilator-free days (β = 1.26, 95% CI 0.71–1.82, *P* < 0.001).

**TABLE 3:** Organ support-free days within 28 days.

Outcomes	N	Crude	*P* value	Adjusted	*P* value
		β (95% CI)		β (95% CI)	
Vasopressor-free days	5650	0.53 (−0.05–1.1)	0.073	1.13 (0.57–1.69)	<0.001
ICU-free days	5650	0.63 (0.07–1.19)	0.027	1.17 (0.63–1.71)	<0.001
Ventilator-free days	5650	0.72 (0.15–1.29)	0.013	1.26 (0.71–1.82)	<0.001

Adjusted for age, sex, hypertension, MAP, temperature, SpO_2_, magnesium, potassium, calcium, hemoglobin, sodium, creatinine, platelets, PT, lactate, anticoagulants, statins, beta blockers, vasoactive agents, ventilation, PCI, CRRT, ECMO, and SOFA.

Abbreviations: β, Beta Coefficient; CI, confidence interval; ICU, intensive care unit; MAP, mean arterial pressure; SpO2, pulse oxygen saturation; PT, prothrombin time; PCI, percutaneous coronary intervention; CRRT, continuous renal replacement therapy; ECMO, extracorporeal membrane oxygenation; SOFA, sequential organ failure assessment.

### Dose–response relationship analysis

Dose-response relationships between magnesium sulfate exposure and mortality outcomes were evaluated across different dosage and duration categories (Tables 4–6). Baseline characteristics across the three dosage groups are presented in Supplementary Table S4. In the analysis stratified by cumulative dose, the medium-dose group (2.0–8.0 g) demonstrated the most substantial survival benefit compared to the low-dose group (< 2.0 g). In multivariable-adjusted models, this dose range was associated with a 64% reduction in 30-day mortality (HR 0.36, 95% CI 0.29–0.45, *P* < 0.001), 63% reduction in 90-day mortality (HR 0.37, 95% CI 0.31–0.45, *P* < 0.001), and 59% reduction in 365-day mortality (HR 0.41, 95% CI 0.35–0.48, *P* < 0.001). In contrast, the high-dose group (≥ 8.0 g) showed no significant risk reduction compared to the reference group for 30-day (adjusted HR 1.00, 95% CI 0.85–1.18, *P* = 0.983), 90-day (HR 1.04, 95% CI 0.90–1.20, *P* = 0.586), or 365-day mortality (HR 1.03, 95% CI 0.91–1.17, *P* = 0.591). To validate the robustness of these findings, we performed 1:1:1 propensity score matching, resulting in 520 patients per group. Baseline characteristics before and after matching are shown in Supplementary Table S5, with standardized mean differences visualized in Supplementary Figure S2. The propensity score-matched analyses yielded consistent results.

**TABLE 4:** 30-day mortality stratified by cumulative dosage and treatment duration of intravenous magnesium sulfate in critically ill patients with AMI.

Subgroups	Total	N (%)	Crude		Adjusted	
			HR (95% CI)	*P* value	HR (95% CI)	*P* value
Dosage (g)						
<2.0	1144	254 (22.2)	1 (Ref)	​	1 (Ref)	​
2.0–8.0	2088	152 (7.3)	0.30 (0.24–0.36)	<0.001	0.36 (0.29–0.45)	<0.001
≥8.0	2418	612 (25.3)	1.14 (0.99–1.32)	0.073	1.00 (0.85–1.18)	0.983
Duration (days)						
<1.08	1883	382 (20.3)	1 (Ref)	​	1 (Ref)	​
1.08–3.21	1868	236 (12.6)	0.60 (0.51–0.70)	<0.001	0.71 (0.60–0.84)	<0.001
≥3.21	1899	400 (21.1)	1.00 (0.87–1.15)	0.982	0.76 (0.65–0.90)	0.001

Adjusted for age, sex, hypertension, MAP, temperature, SpO_2_, magnesium, potassium, calcium, hemoglobin, sodium, creatinine, platelets, PT, lactate, anticoagulants, statins, beta blockers, vasoactive agents, ventilation, PCI, CRRT, ECMO, and SOFA.

Abbreviations: AMI, acute myocardial infarction; HR, hazard ratio; CI, confidence interval; MAP, mean arterial pressure; SpO2, pulse oxygen saturation; PT, prothrombin time; PCI, percutaneous coronary intervention; CRRT, continuous renal replacement therapy; ECMO, extracorporeal membrane oxygenation; SOFA, sequential organ failure assessment.

**TABLE 5:** 60-day mortality stratified by cumulative dosage and treatment duration of intravenous magnesium sulfate in critically ill patients with AM**I**.

Subgroups	Total	N (%)	Crude		Adjusted	
			HR (95% CI)	P value	HR (95% CI)	P value
Dosage (g)						
<2.0	1144	293 (25.6)	1 (Ref)	​	1 (Ref)	​
2.0–8.0	2088	189 (9.1)	0.32 (0.26–0.38)	<0.001	0.38 (0.31–0.47)	<0.001
≥8.0	2418	715 (29.6)	1.17 (1.02–1.34)	0.027	1.05 (0.91–1.22)	0.484
Duration (days)						
<1.08	1883	442 (23.5)	1 (Ref)	​	1 (Ref)	​
1.08–3.21	1868	282 (15.1)	0.61 (0.53–0.71)	<0.001	0.73 (0.63–0.85)	<0.001
≥3.21	1899	473 (24.9)	1.03 (0.90–1.17)	0.666	0.83 (0.72–0.97)	0.017

Adjusted for age, sex, hypertension, MAP, temperature, SpO_2_, magnesium, potassium, calcium, hemoglobin, sodium, creatinine, platelets, PT, lactate, anticoagulants, statins, beta blockers, vasoactive agents, ventilation, PCI, CRRT, ECMO, and SOFA.

Abbreviations: AMI, acute myocardial infarction; HR, hazard ratio; CI, confidence interval; MAP, mean arterial pressure; SpO2, pulse oxygen saturation; PT, prothrombin time; PCI, percutaneous coronary intervention; CRRT, continuous renal replacement therapy; ECMO, extracorporeal membrane oxygenation; SOFA, sequential organ failure assessment.

**TABLE 6:** 365-day mortality stratified by cumulative dosage and treatment duration of intravenous magnesium sulfate in critically ill patients with AM**I**.

Subgroups	Total	N (%)		Crude		Adjusted	
				HR (95% CI)	P value	HR (95% CI)	P value
Dosage (g)							
<2.0	1144	442 (38.6)	1 (Ref)	​	1 (Ref)	​	​
2.0–8.0	2088	302 (14.5)	0.32 (0.28–0.37)	<0.001	0.41 (0.35–0.48)	<0.001	​
≥8.0	2418	987 (40.8)	1.08 (0.96–1.21)	0.185	1.03 (0.91–1.17)	0.591	​
Duration (days)							
<1.08	1883	657 (34.9)	1 (Ref)	​	1 (Ref)	​	​
1.08–3.21	1868	421 (22.5)	0.60 (0.53–0.68)	<0.001	0.73 (0.65–0.83)	<0.001	​
≥3.21	1899	653 (34.4)	0.97 (0.87–1.08)	0.544	0.86 (0.76–0.97)	0.018	​

Adjusted for age, sex, hypertension, MAP, temperature, SpO_2_, magnesium, potassium, calcium, hemoglobin, sodium, creatinine, platelets, PT, lactate, anticoagulants, statins, beta blockers, vasoactive agents, ventilation, PCI, CRRT, ECMO, and SOFA.

Abbreviations: AMI, acute myocardial infarction; HR, hazard ratio; CI, confidence interval; MAP, mean arterial pressure; SpO2, pulse oxygen saturation; PT, prothrombin time; PCI, percutaneous coronary intervention; CRRT, continuous renal replacement therapy; ECMO, extracorporeal membrane oxygenation; SOFA, sequential organ failure assessment.

In the analysis stratified by treatment duration, the medium-duration group (1.08–3.21 days) showed significantly lower mortality risks compared to the short-duration group (<1.08 days), with adjusted hazard ratios of 0.71 (95% CI 0.60–0.84, *P* < 0.001) for 30-day mortality, 0.72 (95% CI 0.62–0.83, *P* < 0.001) for 90-day mortality, and 0.73 (95% CI 0.65–0.83, *P* < 0.001) for 365-day mortality. The long-duration group (≥3.21 days) also exhibited protective effects, though generally of lesser magnitude, with adjusted hazard ratios of 0.76 (95% CI 0.65–0.90, *P* = 0.001) for 30-day mortality, 0.84 (95% CI 0.73–0.98, *P* = 0.022) for 90-day mortality, and 0.86 (95% CI 0.76–0.97, *P* = 0.018) for 365-day mortality.

### Subgroup analysis

**FIGURE 6:**
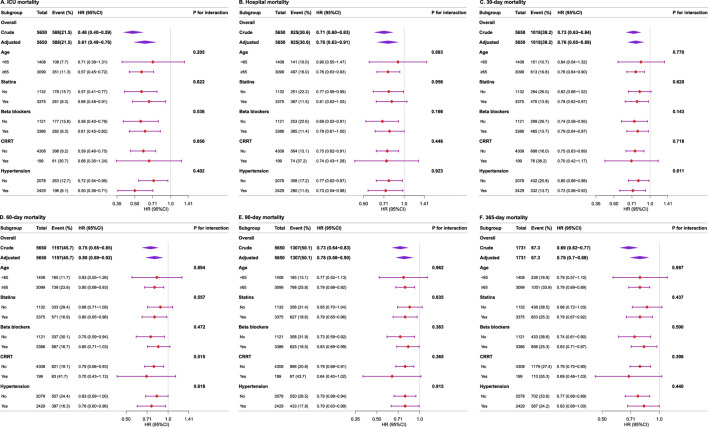
Subgroup analyses of the association between intravenous magnesium sulfate administration and mortality: **(A)** ICU mortality, **(B)** hospital mortality, **(C)** 30-day mortality, **(D)** 60-day mortality, **(E)** 90-day mortality, and **(F)** 365-day mortality.

Subgroup analyses were conducted to evaluate the consistency of the treatment effect of magnesium sulfate administration on ICU mortality across clinically relevant subgroups (Figure 6A). Magnesium sulfate showed consistent protective effects across all pre-specified subgroups, including age strata, statin use, beta-blocker use, CRRT, and hypertension status, with no significant effect modification detected (all P for interaction > 0.05). Similarly, in analyses of all secondary mortality endpoints, the treatment effect remained homogeneous across all subgroups, with no significant interactions identified (all P for interaction > 0.05; Figures 6B–F).

### Sensitivity analysis

**FIGURE 7:**
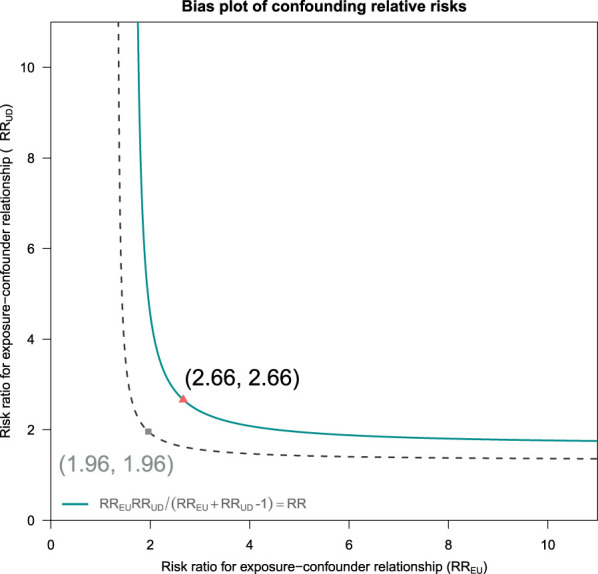
Bias plot for assessing unmeasured confounding.

For the current study, the adjusted hazard ratio (HR) for the association between intravenous magnesium sulfate and ICU mortality was 0.61 (95% CI 0.49–0.76). The E-value for the point estimate was 2.66, and the E-value for the upper confidence limit was 1.96 (Figure 7). This suggests that an unmeasured confounder would need to be associated with both intravenous magnesium sulfate administration and ICU mortality by risk ratios of at least 2.66-fold each, beyond the covariates already adjusted for, to fully explain away the observed protective association.

## Discussion

This study utilized a real-world cohort from the MIMIC-IV database to evaluate the impact of intravenous magnesium sulfate on mortality in critically ill AMI patients. Propensity score matching and multiple sensitivity analyses demonstrated that magnesium sulfate administration was associated with a significant reduction in ICU mortality risk (HR 0.55). This benefit was corroborated by an increase in restricted mean survival time. Quantitative sensitivity analysis suggested that unmeasured confounding is unlikely to fully explain this association (E-value = 2.66). A clear dose-response relationship was observed, with the greatest benefit at a moderate cumulative dose (2.0–8.0 g) and treatment duration (1.08–3.21 days). Subgroup analyses indicated consistent protective effects across all patient strata.

The dose-response relationship observed in our study exhibited a U-shaped pattern. The moderate-dose group (2.0–8.0 g) demonstrated the most significant protective effect, associated with a 64% reduction in 30-day mortality, whereas no additional therapeutic benefit was observed in the high-dose group (≥8.0 g). This finding aligns with established pharmacological principles. Excessively high doses of magnesium sulfate can cause hypermagnesemia, resulting in adverse effects such as muscle weakness, hypotension, bradycardia, and conduction abnormalities (Aal-Hamad et al., 2023; Tangvoraphonkchai and Davenport, 2018). Furthermore, excessive inhibition of calcium channels may impair myocardial contractility, potentially exacerbating the condition in critically ill patients with pre-existing hemodynamic instability (Agarwal et al., 2018). This pattern aligns with the pharmacological principle that interventions with potent physiological effects often exhibit a narrow therapeutic window. Such a 'double-edged sword’ phenomenon is not unique to magnesium; similar non-linear dose-response patterns have been reported for other cardioprotective and neuroprotective agents in acute ischemic settings, where moderate doses confer protection while higher doses lose efficacy or increase harm (Sanches et al., 2021).

Our findings of a U-shaped dose-response relationship help reconcile the seemingly conflicting results of prior clinical trials. Early clinical interest in magnesium was bolstered by the LIMIT-2 trial, which reported a significant mortality reduction with early administration in higher-risk patients (Woods et al., 1992). In contrast, the larger ISIS-4 trial and the MAGIC trial found no benefit using high-dose regimens (approximately 20 g and 19 g, respectively) in broader AMI populations with later administration (Clinical Trial, 1995; Antman, 2002). Notably, these high-dose regimens correspond to our ≥8.0 g subgroup, in which we also observed no survival benefit. Thus, the null results of ISIS-4 and MAGIC are consistent with our high-dose findings, whereas the moderate-dose (2.0–8.0 g) benefit revealed by our dose-response analysis may represent an optimal therapeutic window that was not examined in prior studies. Beyond dosing, differences in patient selection and timing may also contribute to the discrepant outcomes. The LIMIT-2 trial enrolled higher-risk patients and initiated magnesium within 3 h on average after symptom onset, whereas these two high-dose trials included broader populations with later administration (median 4–5 h), often after reperfusion therapy, potentially missing the optimal therapeutic window.

Emerging evidence underscores the importance of magnesium homeostasis in high-risk populations. Hypomagnesemia has been linked to increased mortality in patients with heart failure (Sato et al., 2025). In the general critically ill population, low magnesium levels are correlated with adverse clinical courses, including prolonged mechanical ventilation, extended ICU stay, and increased mortality (Llewellyn and Cutler, 2026; Laddhad et al., 2023). For patients with ST-segment elevation myocardial infarction undergoing primary percutaneous intervention, intravenous magnesium sulfate administered during reperfusion significantly reduces reperfusion arrhythmias, with some evidence suggesting a potential improvement in survival (Szapary et al., 2021). Our study specifically evaluates this intervention in critically ill AMI patients within the ICU setting. The results indicate that intravenous magnesium sulfate therapy may be associated with reduced mortality in this high-risk cohort.

In this cohort, the rate of percutaneous coronary intervention (PCI) was 19.0%, reflecting the critical illness and high comorbidity burden of these patients, including heart failure (52.1%), chronic kidney disease (30.1%), mechanical ventilation (40.9%), and vasoactive support (40.4%). Many were unsuitable for PCI due to clinical instability or underwent CABG (29.9%) instead. Importantly, PCI status was adjusted for in all multivariable and propensity score models, ensuring that the estimated magnesium effect is independent of revascularization. Despite the widespread adoption of PCI during the study period (2008–2022), magnesium sulfate may serve as a valuable adjunctive therapy in this high-risk population by mitigating reperfusion injury and stabilizing hemodynamics, complementing mechanical revascularization where feasible (Rao et al., 2025).

The cardioprotective effects of magnesium sulfate in AMI are mediated through multiple physiological pathways. First, as a natural calcium channel antagonist, magnesium competes with calcium influx, mitigating intracellular calcium overload during ischemia-reperfusion, thereby protecting cardiomyocytes from necrosis and apoptosis (Lai et al., 2024; Houston, 2011). Second, magnesium exerts antioxidant effects by stabilizing cell membranes and enhancing antioxidant enzyme activity, reducing oxidative stress and lipid peroxidation (Shechter et al., 2000; Kumar et al., 2024; Ashique et al., 2023). Third, it possesses antiarrhythmic properties by stabilizing membrane potentials and prolonging the effective refractory period, significantly reducing the incidence of life-threatening ventricular arrhythmias (Negru et al., 2022; Sleeswijk et al., 2008). Fourth, magnesium induces vasodilation by inhibiting calcium influx in vascular smooth muscle and promoting prostacyclin release, which improves coronary perfusion and reduces afterload (Shechter, 2003; Kolte et al., 2014). Finally, as an essential cofactor in ATP synthesis, magnesium supports cellular energy metabolism, enhancing the myocardium’s tolerance to ischemic injury (Patni et al., 2022).

Several limitations should be considered. First, the retrospective observational design precludes causal inference, and residual confounding may persist despite statistical adjustments. Our analysis relied on baseline laboratory values and did not account for longitudinal physiological changes during ICU stay, which may influence patient outcomes. Second, selection bias cannot be excluded, as the decision to administer treatment may have been influenced by clinical factors not recorded in the database (Ding et al., 2025). Third, detailed data on the magnesium sulfate administration regimen and the clinical indications for its use were limited, and information on arrhythmias, reperfusion timing, and infarct severity was not available in the database, precluding a granular analysis of how these factors may have influenced outcomes. Fourth, the study focused primarily on mortality, lacking data on other clinical endpoints such as heart failure or detailed safety profiles. Furthermore, the use of a single-center database may limit the generalizability of the findings.

## Conclusion

Intravenous magnesium sulfate may serve as a beneficial adjunctive therapy for critically ill AMI patients in the ICU. A treatment regimen utilizing a moderate dose over an appropriate duration warrants careful consideration in clinical practice and further investigation in prospective studies.

## Data Availability

The data underlying this article were accessed from PhysioNet (https://physionet.org/content/mimiciv/, version 3.1). The derived data generated in this research will be shared on reasonable request to the corresponding author with permission from PhysioNet.
