# Supplementary material for: The impact of magnesium sulfate administration on outcomes in ICU patients with acute myocardial infarction: a retrospective cohort study
Source: Front Pharmacol. 2026 May 25;17:1776926. doi: 10.3389/fphar.2026.1776926 (PMC13243276; doi:10.3389/fphar.2026.1776926)
Supplement: Supplementary file 1 [file Table1.docx]

**The Impact of Magnesium Sulfate Administration on Outcomes in ICU Patients with Acute Myocardial Infarction: A Retrospective Cohort Study**

**Supplementary materials**

**Supplementary Table 1.** Baseline Characteristics of Critically Ill Patients with AMI

**Supplementary Table 2.** Missing Data Distribution in the Study Cohort

**Supplementary Table 3.** Restricted Mean Survival Time Differences for ICU and Hospital Mortality

**Supplementary Table 4.** Baseline Characteristics of Critically Ill Patients with AMI Stratified by Intravenous Magnesium Sulfate Dosage

**Supplementary Table 5.** Baseline Characteristics of Critically Ill AMI Patients Stratified by Intravenous Magnesium Sulfate Dosage Before and After Propensity Score Matching

**Supplementary Figure 1.** Receiver operating characteristic (ROC) curve of the propensity score-matched (PSM) model for predicting ICU mortality

**Supplementary Figure 2.** Balance of covariates across three intravenous magnesium sulfate dosage groups in critically ill AMI patients before and after propensity score matching for ICU mortality assessment

**Supplementary Table 1.** Baseline Characteristics of Critically Ill Patients with AMI

| **Variables** | **Total (n = 5650)** | **Magnesium sulfate use** | | ***P* value** |
| --- | --- | --- | --- | --- |
|  |  | **No (n = 1143)** | **Yes (n = 4507)** |  |
| Age (years) | 71.1 ± 12.9 | 73.4 ± 13.5 | 70.6 ± 12.7 | < 0.001 |
| Male (n, %) | 3654 (64.7) | 726 (63.5) | 2928 (65.0) | 0.360 |
| Heart rate (bpm) | 82.9 ± 14.6 | 80.7 ± 14.8 | 83.5 ± 14.4 | < 0.001 |
| MAP (mmHg) | 77.5 ± 9.8 | 77.5 ± 11.1 | 77.4 ± 9.5 | 0.784 |
| Respiratory rate (bpm) | 19.5 ± 3.6 | 19.8 ± 3.7 | 19.5 ± 3.5 | 0.005 |
| Temperature (°C) | 36.8 ± 0.5 | 36.7 ± 0.5 | 36.8 ± 0.5 | < 0.001 |
| SpO2 (%) | 96.9 ± 2.0 | 96.6 ± 1.8 | 97.0 ± 2.1 | < 0.001 |
| Weight (Kg) | 83.1 ± 21.4 | 81.8 ± 22.2 | 83.4 ± 21.2 | 0.020 |
| Platelets (10^9^/L) | 184.2 ± 86.6 | 199.4 ± 88.5 | 180.3 ± 85.6 | < 0.001 |
| WBC (10^9^/L) | 13.8 (10.4, 18.6) | 11.9 (9.1, 16.0) | 14.4 (10.9, 19.2) | < 0.001 |
| Anion gap (mmol/L) | 16.7 ± 5.2 | 17.7 ± 5.2 | 16.5 ± 5.2 | < 0.001 |
| Calcium (mg/dL) | 8.2 ± 0.8 | 8.4 ± 0.8 | 8.1 ± 0.8 | < 0.001 |
| Hemoglobin (g/dL) | 10.1 ± 2.3 | 10.3 ± 2.4 | 10.0 ± 2.3 | < 0.001 |
| Creatinine (mg/ dL) | 1.2 (0.9, 1.9) | 1.5 (1.0, 2.8) | 1.2 (0.9, 1.8) | < 0.001 |
| Sodium (mmol/L) | 136.3 ± 4.7 | 136.5 ± 5.1 | 136.3 ± 4.6 | 0.105 |
| Potassium (mmol/L) | 4.8 ± 0.8 | 4.8 ± 0.9 | 4.7 ± 0.8 | < 0.001 |
| PT (s) | 16.9 ± 10.2 | 17.0 ± 11.5 | 16.9 ± 9.8 | 0.699 |
| Lactate (mmol/L) | 2.3 (1.8, 3.1) | 2.3 (1.8, 2.9) | 2.3 (1.8, 3.2) | < 0.001 |
| Magnesium (mmol/L) | 2.1 ± 0.5 | 2.2 ± 0.4 | 2.1 ± 0.5 | < 0.001 |
| CHF (n, %) | 2942 (52.1) | 663 (58.0) | 2279 (50.6) | < 0.001 |
| Hypertension (n, %) | 2932 (51.9) | 503 (44.0) | 2429 (53.9) | < 0.001 |
| Diabetes (n, %) | 2415 (42.7) | 512 (44.8) | 1903 (42.2) | 0.117 |
| CKD (n, %) | 1702 (30.1) | 471 (41.2) | 1231 (27.3) | < 0.001 |
| SAPS II (scores) | 39.5 ± 13.8 | 39.1 ± 14.6 | 39.6 ± 13.6 | 0.231 |
| OASIS (scores) | 33.6 ± 9.5 | 31.8 ± 9.4 | 34.1 ± 9.5 | < 0.001 |
| SOFA (scores) | 5.1 ± 3.3 | 4.5 ± 3.4 | 5.3 ± 3.3 | < 0.001 |
| APACHE II (scores) | 18.9 ± 7.6 | 18.4 ± 7.9 | 19.1 ± 7.5 | 0.004 |
| CRRT (n, %) | 298 ( 5.3) | 99 (8.7) | 199 (4.4) | < 0.001 |
| Ventilation (n, %) | 2310 (40.9) | 228 (19.9) | 2082 (46.2) | < 0.001 |
| Vasoactive agents (n, %) | 2284 (40.4) | 251 (22.0) | 2033 (45.1) | < 0.001 |
| ECMO (n, %) | 22 ( 0.4) | 3 (0.3) | 19 (0.4) | 0.598 |
| IABP (n, %) | 284 ( 5.0) | 25 (2.2) | 259 (5.7) | < 0.001 |
| PCI (n, %) | 1072 (19.0) | 282 (24.7) | 790 (17.5) | < 0.001 |
| CABG (n, %) | 1692 (29.9) | 86 (7.5) | 1606 (35.6) | < 0.001 |
| Beta blockers (n, %) | 4163 (73.7) | 777 (68.0) | 3386 (75.1) | < 0.001 |
| Anticoagulants (n, %) | 4854 (85.9) | 1023 (89.5) | 3831 (85.0) | < 0.001 |
| Statins (n, %) | 3991 (70.6) | 616 (53.9) | 3375 (74.9) | < 0.001 |
| **Antiplatelet drugs** (n, %) | 4696 (83.1) | 806 (70.5) | 3890 (86.3) | < 0.001 |
| Vasopressor-free days within 28-days | 22.2 ± 10.8 | 21.6 ± 11.6 | 22.4 ± 10.7 | 0.039 |
| ICU-free days within 28-days | 19.6 ± 10.5 | 19.6 ± 10.8 | 19.6 ± 10.5 | 0.994 |
| Ventilator-free days wihtin 28-days | 22.2 ± 10.7 | 21.6 ± 11.5 | 22.3 ± 10.5 | 0.037 |
| Length of ICU Stay (days) | 2.6 (1.6, 4.8) | 2.1 (1.4, 3.5) | 2.9 (1.6, 5.2) | < 0.001 |
| Length of hospital Stay (days) | 8.5 (5.2, 13.6) | 6.8 (3.7, 12.0) | 8.9 (5.8, 13.9) | < 0.001 |
| ICU mortality (n, %) | 588 (10.4) | 129 (11.3) | 459 (10.2) | 0.276 |
| Hospital mortality (n, %) | 825 (14.6) | 187 (16.4) | 638 (14.2) | 0.059 |
| 30-day mortality (n, %) | 1018 (18.0) | 254 (22.2) | 764 (17.0) | < 0.001 |
| 60-day mortality (n, %) | 1197 (21.2) | 293 (25.6) | 904 (20.1) | < 0.001 |
| 90-day mortality (n, %) | 1307 (23.1) | 324 (28.3) | 983 (21.8) | < 0.001 |
| 365-day mortality (n, %) | 1731 (30.6) | 442 (38.7) | 1289 (28.6) | < 0.001 |

Abbreviations: AMI, Acute Myocardial Infarction; MAP, mean arterial pressure; SpO2, percutaneous arterial oxygen saturation; WBC, white blood cell count; PT, prothrombin time; CHF, congestive heart failure; CKD, chronic kidney disease; SAPS II, simplified acute physiology score II; OASIS, oxford acute severity of illness score; SOFA, sequential organ failure assessment; APACHE II, Acute Physiology and Chronic Health Evaluation II; CRRT, continuous renal replacement therapy; ECMO, extracorporeal membrane oxygenation; IABP; intra-aortic balloon pump; PCI, **Percutaneous Coronary Intervention;** CABG,coronary artery bypass grafting; ICU, intensive care unit.

**Supplementary Table 2.** Missing Data Distribution in the Study Cohort

| **Variables** | ****Number of missing variables**** | **Missing data** |
| --- | --- | --- |
| MAP | 7 | 0.12% |
| Temperature | 248 | 4.39% |
| SpO_2_ | 7 | 0.12% |
| Magnesium | 241 | 4.27% |
| Potassium | 18 | 0.32% |
| Calcium | 522 | 9.24% |
| Hemoglobin | 17 | 0.30% |
| Sodium | 15 | 0.27% |
| Creatinine | 14 | 0.25% |
| Platelets | 16 | 0.28% |
| PT | 203 | 3.59% |
| Lactate | 2208 | 39.08% |

**Supplementary Table 3.** Restricted Mean Survival Time Differences for ICU and Hospital Mortality

| **Model** | **Time** | **HR/RMSTd (95% CI)** | ***P* Value** |
| --- | --- | --- | --- |
| **ICU mortality** | | | |
|  | 14-day | 1.638 (0.970-2.305) | < 0.001 |
|  | 30-day | 5.270 (2.630-7.911) | < 0.001 |
| **Hospital mortality** | | | |
|  | 14-day | 0.509 (0.257-0.760) | < 0.001 |
|  | 30-day | 1.463 (0.522-2.404) | 0.002 |

**Supplementary Table 4.** Baseline Characteristics of Critically Ill Patients with AMI Stratified by Intravenous Magnesium Sulfate Dosage

| **Variables** | **Total (n = 5650)** | **Intravenous Magnesium Sulfate Dosage (g)** | | | ***P***  **Value** |
| --- | --- | --- | --- | --- | --- |
|  |  | **< 2.0** | **(2.0, 8.0)** | **≥ 8.0** |  |
| Participants (n) | 5650 | 1144 | 2088 | 2418 |  |
| Age (years) | 71.1 ± 12.9 | 73.4 ± 13.5 | 69.8 ± 11.6 | 71.2 ± 13.6 | < 0.001 |
| Male (n, %) | 3654 (64.7) | 727 (63.5) | 1474 (70.6) | 1453 (60.1) | < 0.001 |
| Heart rate (bpm) | 82.9 ± 14.6 | 80.7 ± 14.8 | 82.4 ± 11.6 | 84.4 ± 16.4 | < 0.001 |
| MAP (mmHg) | 77.5 ± 9.8 | 77.5 ± 11.1 | 75.4 ± 8.0 | 79.2 ± 10.3 | < 0.001 |
| Respiratory rate (bpm) | 19.5 ± 3.6 | 19.8 ± 3.7 | 18.5 ± 2.9 | 20.3 ± 3.8 | < 0.001 |
| Temperature (°C) | 36.8 ± 0.5 | 36.7 ± 0.5 | 36.8 ± 0.5 | 36.8 ± 0.6 | < 0.001 |
| SpO2 (%) | 96.9 ± 2.0 | 96.6 ± 1.8 | 97.4 ± 1.8 | 96.6 ± 2.2 | < 0.001 |
| Weight (Kg) | 83.1 ± 21.4 | 81.7 ± 22.2 | 85.4 ± 19.9 | 81.7 ± 22.1 | < 0.001 |
| Platelets (10^9^/L) | 184.2 ± 86.6 | 199.4 ± 88.5 | 167.2 ± 76.3 | 191.6 ± 91.5 | < 0.001 |
| WBC (10^9^/L) | 13.8 (10.4, 18.6) | 11.9 (9.1, 16.0) | 15.1 (11.6, 19.5) | 13.7 (10.3, 18.8) | < 0.001 |
| Anion gap (mmol/L) | 16.7 ± 5.2 | 17.7 ± 5.2 | 14.4 ± 4.2 | 18.2 ± 5.3 | < 0.001 |
| Calcium (mg/dL) | 8.2 ± 0.8 | 8.4 ± 0.8 | 8.2 ± 0.7 | 8.1 ± 0.8 | < 0.001 |
| Hemoglobin (g/dL) | 10.1 ± 2.3 | 10.3 ± 2.4 | 9.6 ± 2.0 | 10.4 ± 2.4 | < 0.001 |
| Creatinine (mg/ dL) | 1.2 (0.9, 1.9) | 1.5 (1.0, 2.8) | 1.0 (0.8, 1.4) | 1.3 (1.0, 2.1) | < 0.001 |
| Sodium (mmol/L) | 136.3 ± 4.7 | 136.5 ± 5.1 | 136.4 ± 3.4 | 136.2 ± 5.5 | 0.095 |
| Potassium (mmol/L) | 4.8 ± 0.8 | 4.8 ± 0.9 | 4.7 ± 0.6 | 4.8 ± 0.9 | < 0.001 |
| PT (s) | 16.9 ± 10.2 | 17.0 ± 11.5 | 16.7 ± 7.9 | 17.1 ± 11.2 | 0.431 |
| Lactate (mmol/L) | 2.3 (1.8, 3.1) | 2.3 (1.8, 2.9) | 2.4 (1.9, 3.2) | 2.3 (1.7, 3.2) | < 0.001 |
| Magnesium (mmol/L) | 2.1 ± 0.5 | 2.2 ± 0.4 | 2.3 ± 0.6 | 2.0 ± 0.4 | < 0.001 |
| CHF (n, %) | 2942 (52.1) | 664 (58) | 846 (40.5) | 1432 (59.2) | < 0.001 |
| Hypertension (n, %) | 2932 (51.9) | 503 (44) | 1217 (58.3) | 1212 (50.1) | < 0.001 |
| Diabetes (n, %) | 2415 (42.7) | 512 (44.8) | 924 (44.3) | 979 (40.5) | 0.012 |
| CKD (n, %) | 1702 (30.1) | 472 (41.3) | 507 (24.3) | 723 (29.9) | < 0.001 |
| SAPS II (scores) | 39.5 ± 13.8 | 39.1 ± 14.6 | 38.6 ± 12.8 | 40.5 ± 14.2 | < 0.001 |
| OASIS (scores) | 33.6 ± 9.5 | 31.8 ± 9.4 | 33.1 ± 8.6 | 35.0 ± 10.1 | < 0.001 |
| SOFA (scores) | 5.1 ± 3.3 | 4.5 ± 3.4 | 5.4 ± 2.9 | 5.2 ± 3.6 | < 0.001 |
| APACHE II (scores) | 18.9 ± 7.6 | 18.4 ± 7.9 | 18.6 ± 6.9 | 19.5 ± 8.1 | < 0.001 |
| CRRT (n, %) | 298 ( 5.3) | 99 (8.7) | 53 (2.5) | 146 (6.0) | < 0.001 |
| Ventilation (n, %) | 2310 (40.9) | 228 (19.9) | 1197 (57.3) | 885 (36.6) | < 0.001 |
| Vasoactive agents (n, %) | 2284 (40.4) | 252 (22) | 1123 (53.8) | 909 (37.6) | < 0.001 |
| ECMO (n, %) | 22 ( 0.4) | 3 (0.3) | 3 (0.1) | 16 (0.7) | 0.015 |
| IABP (n, %) | 284 ( 5.0) | 25 (2.2) | 80 (3.8) | 179 (7.4) | < 0.001 |
| PCI (n, %) | 1072 (19.0) | 283 (24.7) | 219 (10.5) | 570 (23.6) | < 0.001 |
| CABG (n, %) | 1692 (29.9) | 86 (7.5) | 1418 (67.9) | 188 (7.8) | < 0.001 |
| Beta blockers (n, %) | 4163 (73.7) | 778 (68.0) | 1730 (82.9) | 1655 (68.4) | < 0.001 |
| Anticoagulants (n, %) | 4854 (85.9) | 1024 (89.5) | 1509 (72.3) | 2321 (96) | < 0.001 |
| Statins (n, %) | 3991 (70.6) | 617 (53.9) | 1679 (80.4) | 1695 (70.1) | < 0.001 |
| **Antiplatelet drugs** (n, %) | 4696 (83.1) | 807 (70.5) | 1922 (92.0) | 1967 (81.3) | < 0.001 |
| Vasopressor-free days within 28-days | 22.2 ± 10.8 | 21.6 ± 11.6 | 25.2 ± 7.6 | 19.9 ± 12.2 | < 0.001 |
| ICU-free days within 28-days | 19.6 ± 10.5 | 19.6 ± 10.7 | 22.6 ± 8.1 | 17.1 ± 11.5 | < 0.001 |
| Ventilator-free days wihtin 28-days | 22.2 ± 10.7 | 21.6 ± 11.5 | 25.2 ± 7.4 | 19.8 ± 12.0 | < 0.001 |
| Length of ICU Stay (days) | 2.6 (1.6, 4.8) | 2.1 (1.4, 3.5) | 2.3 (1.3, 4.1) | 3.2 (1.9, 6.3) | < 0.001 |
| Length of hospital Stay (days) | 8.5 (5.2, 13.6) | 6.8 (3.7, 11.9) | 8.9 (6.4, 12.5) | 8.8 (4.9, 15.6) | < 0.001 |
| ICU mortality (n, %) | 588 (10.4) | 129 (11.3) | 96 (4.6) | 363 (15.0) | < 0.001 |
| Hospital mortality (n, %) | 825 (14.6) | 187 (16.3) | 122 (5.8) | 516 (21.3) | < 0.001 |
| 30-day mortality (n, %) | 1018 (18.0) | 254 (22.2) | 152 (7.3) | 612 (25.3) | < 0.001 |
| 60-day mortality (n, %) | 1197 (21.2) | 293 (25.6) | 189 (9.1) | 715 (29.6) | < 0.001 |
| 90-day mortality (n, %) | 1307 (23.1) | 324 (28.3) | 207 (9.9) | 776 (32.1) | < 0.001 |
| 365-day mortality (n, %) | 1731 (30.6) | 442 (38.6) | 302 (14.5) | 987 (40.8) | < 0.001 |

Abbreviations: AMI, Acute Myocardial Infarction; MAP, mean arterial pressure; SpO2, percutaneous arterial oxygen saturation; WBC, white blood cell count; PT, prothrombin time; CHF, congestive heart failure; CKD, chronic kidney disease; SAPS II, simplified acute physiology score II; OASIS, oxford acute severity of illness score; SOFA, sequential organ failure assessment; APACHE II, Acute Physiology and Chronic Health Evaluation II; CRRT, continuous renal replacement therapy; ECMO, extracorporeal membrane oxygenation; IABP; intra-aortic balloon pump; PCI, **Percutaneous Coronary Intervention;** CABG,coronary artery bypass grafting; ICU, intensive care unit.

**Supplementary Table 5.** Baseline Characteristics of Critically Ill AMI Patients Stratified by Intravenous Magnesium Sulfate Dosage Before and After Propensity Score Matching

| **Variables** | **Before propensity score matching** | | | |  | **After propensity score matching** | | | |
| --- | --- | --- | --- | --- | --- | --- | --- | --- | --- |
|  | **Intravenous Magnesium Sulfate Dosage (g)** | | | **SMD** |  | **Intravenous Magnesium Sulfate Dosage (g)** | | | **SMD** |
|  | **< 2.0** | **(2.0, 8.0)** | **≥ 8.0** |  |  | **< 2.0** | **(2.0, 8.0)** | **≥ 8.0** |  |
| Participants (n) | 1144 | 2088 | 2418 |  |  | 520 | 520 | 520 |  |
| Age (years) | 73.39 (13.54) | 69.78 (11.58) | 71.24 (13.60) | 0.187 |  | 71.59 (13.94) | 72.46 (12.82) | 73.20 (13.27) | 0.080 |
| Male (n, %) | 727 (63.55) | 1474 (70.59) | 1453 (60.09) | 0.148 |  | 335 (64.42) | 320 (61.54) | 330 (63.46) | 0.040 |
| Hypertension (n, %) | 503 (43.97) | 1217 (58.29) | 1212 (50.12) | 0.192 |  | 255 (49.04) | 271 (52.12) | 236 (45.38) | 0.090 |
| MAP (mmHg) | 77.52 (11.11) | 75.39 (8.00) | 79.21 (10.31) | 0.264 |  | 77.62 (10.82) | 77.35 (9.85) | 78.05 (9.84) | 0.046 |
| Temperature (℃) | 36.74 (0.49) | 36.75 (0.47) | 36.84 (0.59) | 0.130 |  | 36.75 (0.53) | 36.79 (0.54) | 36.75 (0.62) | 0.056 |
| SpO_2_ (%) | 96.63 (1.81) | 97.39 (1.78) | 96.62 (2.22) | 0.271 |  | 96.82 (1.78) | 96.70 (2.36) | 96.78 (1.80) | 0.040 |
| Magnesium (mmol/L) | 2.18 (0.36) | 2.28 (0.57) | 1.97 (0.42) | 0.460 |  | 2.13 (0.33) | 2.02 (0.48) | 2.10 (0.39) | 0.175 |
| Potassium (mmol/L) | 4.84 (0.93) | 4.66 (0.64) | 4.79 (0.93) | 0.147 |  | 4.77 (0.85) | 4.68 (0.80) | 4.76 (0.86) | 0.072 |
| Calcium (mmol/L) | 8.36 (0.77) | 8.22 (0.67) | 8.08 (0.85) | 0.241 |  | 8.26 (0.84) | 8.19 (0.77) | 8.35 (0.72) | 0.136 |
| Hemoglobin (g/dl) | 10.33 (2.44) | 9.62 (1.97) | 10.36 (2.44) | 0.221 |  | 10.36 (2.56) | 10.37 (2.22) | 10.41 (2.37) | 0.015 |
| Sodium (mmol/L) | 136.52 (5.11) | 136.37 (3.40) | 136.17 (5.48) | 0.048 |  | 136.68 (4.88) | 136.54 (4.29) | 136.26 (5.31) | 0.056 |
| Creatinine (mg/dL) | 2.44 (2.61) | 1.44 (1.43) | 1.87 (1.70) | 0.336 |  | 1.88 (1.53) | 1.85 (1.91) | 2.05 (2.10) | 0.068 |
| Platelets (10^9^/L) | 199.38 (88.47) | 167.18 (76.26) | 191.63 (91.53) | 0.255 |  | 194.65 (90.76) | 197.16 (87.21) | 200.67 (95.33) | 0.044 |
| PT (s) | 17.03 (11.51) | 16.70 (7.89) | 17.08 (11.20) | 0.026 |  | 16.64 (10.93) | 16.79 (9.66) | 17.24 (12.52) | 0.035 |
| Lactate (mmol/L) | 2.65 (1.74) | 2.82 (1.64) | 3.08 (2.67) | 0.136 |  | 2.74 (1.89) | 2.82 (2.05) | 2.78 (2.14) | 0.029 |
| Anticoagulants (n, %) | 1024 (89.51) | 1509 (72.27) | 2321 (95.99) | 0.462 |  | 457 (87.88) | 504 (96.92) | 494 (95.00) | 0.233 |
| Statins (n, %) | 617 (53.93) | 1679 (80.41) | 1695 (70.10) | 0.389 |  | 338 (65.00) | 319 (61.35) | 326 (62.69) | 0.051 |
| Beta blockers (n, %) | 778 (68.01) | 1730 (82.85) | 1655 (68.44) | 0.233 |  | 361 (69.42) | 391 (75.19) | 376 (72.31) | 0.086 |
| Vasoactive agents (n, %) | 252 (22.03) | 1123 (53.78) | 909 (37.59) | 0.456 |  | 154 (29.62) | 158 (30.38) | 130 (25.00) | 0.080 |
| Ventilation (n, %) | 228 (19.93) | 1197 (57.33) | 885 (36.60) | 0.544 |  | 150 (28.85) | 144 (27.69) | 142 (27.31) | 0.023 |
| PCI (n, %) | 283 (24.74) | 219 (10.49) | 570 (23.57) | 0.254 |  | 115 (22.12) | 122 (23.46) | 145 (27.88) | 0.089 |
| CRRT (n, %) | 99 ( 8.65) | 53 ( 2.54) | 146 ( 6.04) | 0.181 |  | 32 ( 6.15) | 29 ( 5.58) | 31 ( 5.96) | 0.016 |
| ECMO (n, %) | 3 ( 0.26) | 3 ( 0.14) | 16 ( 0.66) | 0.056 |  | 1 ( 0.19) | 2 ( 0.38) | 3 ( 0.58) | 0.042 |
| SOFA (score) | 4.46 (3.36) | 5.39 (2.90) | 5.22 (3.62) | 0.188 |  | 4.66 (3.70) | 4.78 (3.39) | 4.48 (3.30) | 0.060 |

An absolute SMD < 10 % was considered to support the assumption of a balance between the groups. Data are presented as medians [interquartile ranges], mean [SD] or as numbers (percentages).

Abbreviations: AMI, Acute Myocardial Infarction; **ICU, I**ntensive Care Unit; SMD, Standardized Mean Difference; MAP, mean arterial pressure; SpO_2_, Pulse Oxygen Saturation; PT, Prothrombin Time; PCI, Percutaneous Coronary Intervention; CRRT, Continuous Renal Replacement Therapy; ECMO, Extracorporeal ane Oxygenation; SOFA, Sequential Organ Failure Assessment.


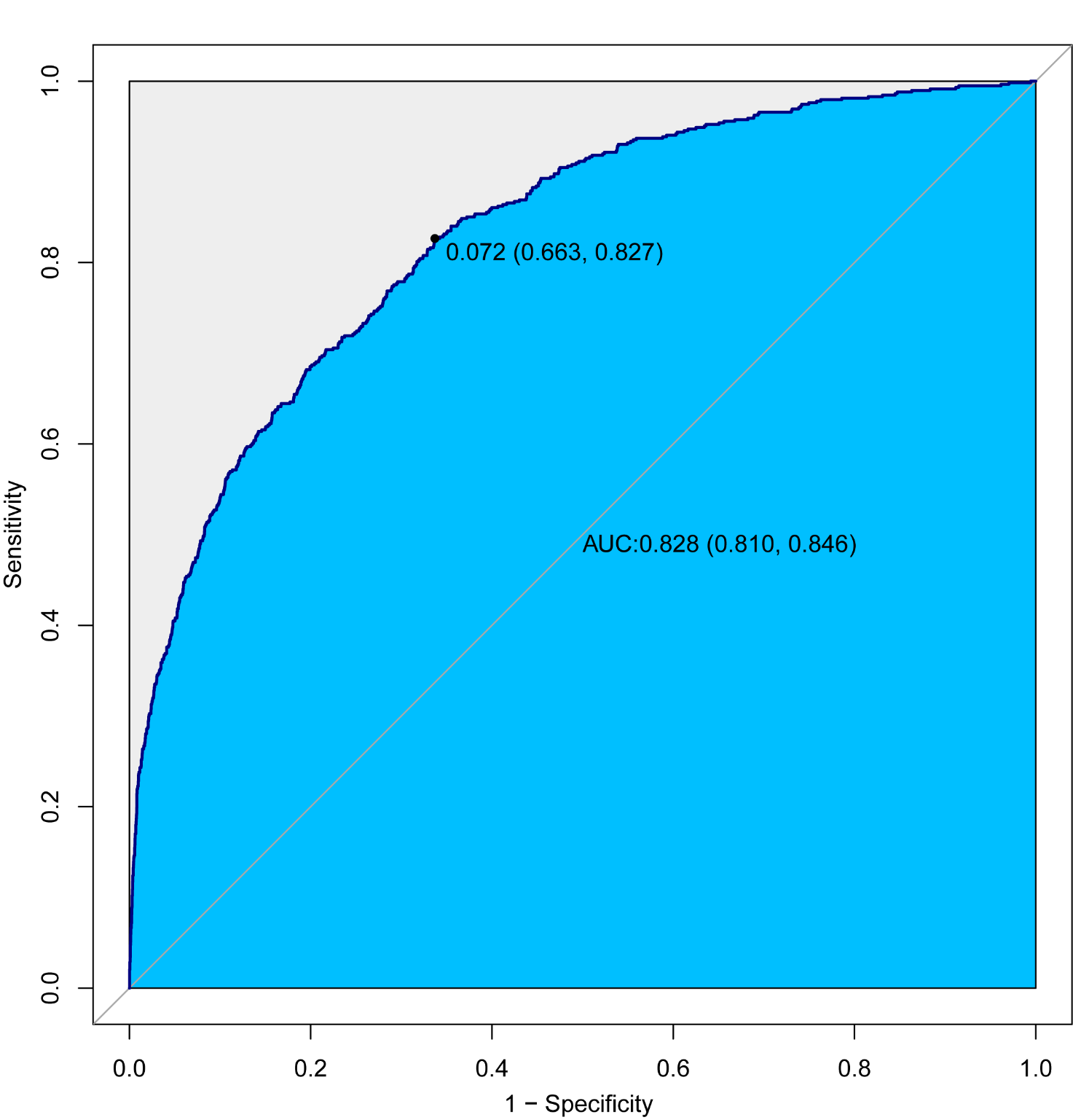


**Supplementary Figure 1.** Receiver operating characteristic (ROC) curve of the propensity score-matched (PSM) model for predicting ICU mortality


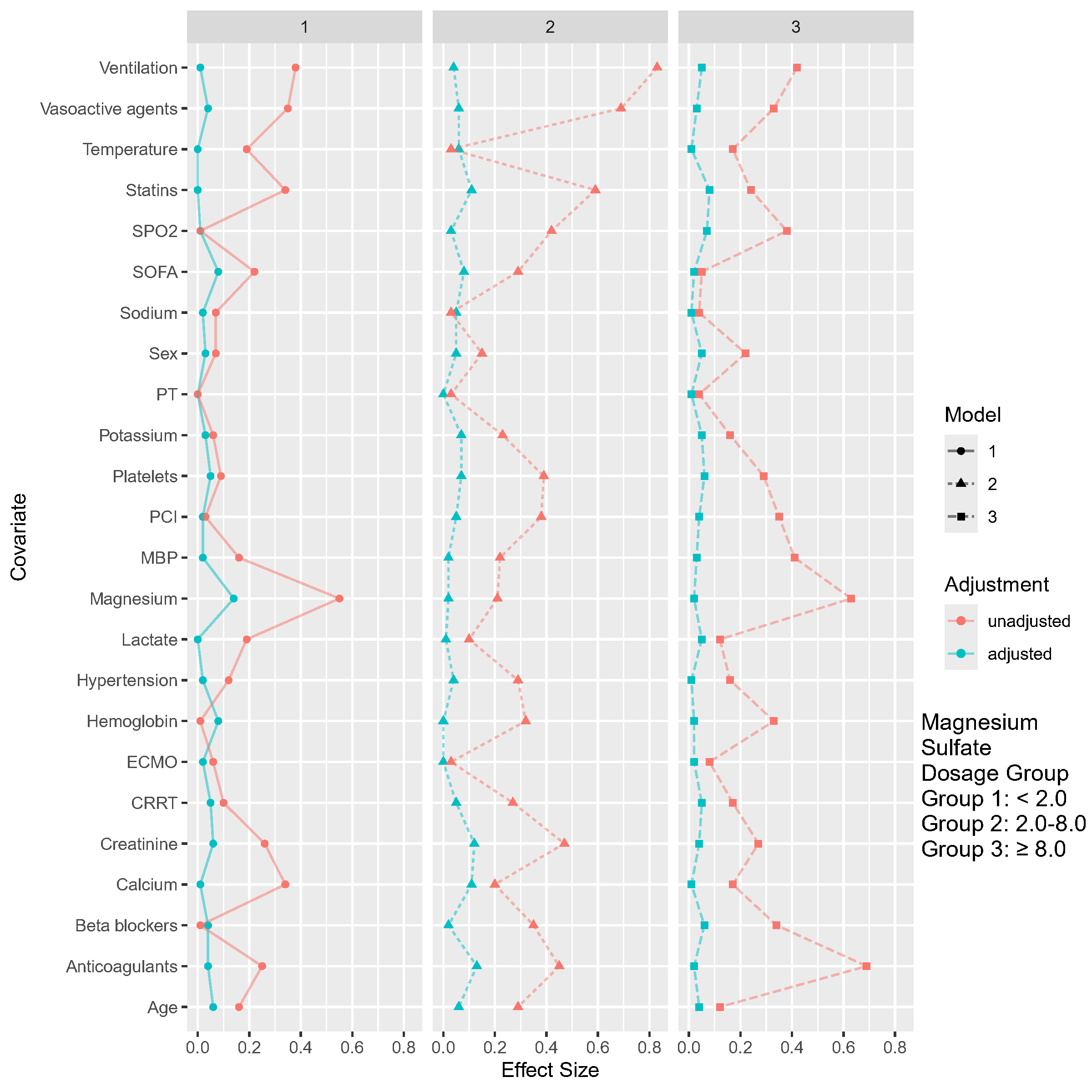


**Supplementary Figure 2.** Balance of covariates across three intravenous magnesium sulfate dosage groups in critically ill AMI patients before and after propensity score matching for ICU mortality assessment
